# Overexpression of Annexin A2 promotes proliferation by forming a Glypican 1/c-Myc positive feedback loop: prognostic significance in human glioma

**DOI:** 10.1038/s41419-021-03547-5

**Published:** 2021-03-12

**Authors:** Xin Li, Shengdan Nie, Ziyang Lv, Lingran Ma, Yuxi Song, Zhongxu Hu, Xin Hu, Zhiqiang Liu, Gaoya Zhou, Zhijie Dai, Tao Song, Jiajia Liu, Shan Wang

**Affiliations:** 1 Department of Neurosurgery, Xiang-Ya Hospital, Central South University, Changsha, China; 2 Department of Pharmaceutical Engineering, College of Chemistry and Chemical Engineering, Central South University, Changsha, China; 3 Institute of Clinical Medicine, Hunan Provincial People’s Hospital, The First Affiliated Hospital of Hunan Normal University, Changsha, China; 4 Department of Neurology, Brain Hospital of Hunan Province, Changsha, China; 5 Department of Institute of Metabolism and Endocrinology, Second Xiang-Ya Hospital, Central South University, Changsha, China

**Keywords:** CNS cancer, Prognostic markers

## Abstract

In order to set up a reliable prediction system for the tumor grade and prognosis in glioma patients, we clarify the complicated crosstalk of Annexin A2 (ANXA2) with Glypican 1 (GPC1) and demonstrate whether combined indexes of ANXA2 and GPC1 could improve the prognostic evaluation for glioma patients. We found that ANXA2-induced glioma cell proliferation in a c-Myc-dependent manner. ANXA2 increased the expression of GPC1 via c-Myc and the upregulated GPC1 further promoted the c-Myc level, forming a positive feedback loop, which eventually led to enhanced proliferation of glioma cells. Both mRNA and protein levels of ANXA2 were upregulated in glioma tissues and coincided with the overexpression of GPC1. Besides, we utilized tissue microarrays (TMAs) and immunohistochemistry to demonstrate that glioma patients with both high expression of ANXA2 and GPC1 tended to have higher rate of tumor recurrence and shorter overall survival (OS). In conclusion, the overexpression of ANXA2 promotes proliferation of glioma cells by forming a GPC1/c-Myc positive feedback loop, and ANXA2 together with its downstream target GPC1 could be a potential “combination biomarker” for predicting prognosis of glioma patients.

## Introduction

Glioma, with varying grades of malignancy, is the most common primary brain tumor^1,2^. Malignant glioma cells are characterized by strong proliferative, migratory, and invasive capacities^3^, which severely constrain the therapeutic effect and result in a poor prognosis^4^. Although researchers have identified various proliferation-associated biomarkers as prognostic indicators for glioma patients^5–7^, such as Par6, chromobox protein homolog 3 (CBX3) and nucleolar and spindle-associated protein 1 (NUSAP1), none of them has been routinely applied in clinical practice. Therefore, understanding the critical mechanisms underlying glioma cell proliferation will help establish a reliable prediction system for tumor grade and prognosis in glioma patients, which can provide guidance for subsequent interventions.

Glypicans (GPCs), a family of heparan sulfate proteoglycans (HSPGs), are anchored to the external surface of the plasma membrane by a glycosylphosphatidylinositol anchor^8–10^. Six members of this family (GPC1–6) have been identified^11,12^. GPCs have been suggested to act as biomarkers for tumor progression, such as GPC3 for hepatocellular carcinoma^13,14^, GPC1 for pancreatic ductal adenocarcinoma^15^, and GPC5 and GPC1 for prostate cancer^16,17^. GPC1 is universally overexpressed in human glioma^18,19^. It was reported that overexpression of GPC1 in U87 glioma cells enhanced FGF-2-stimulated proliferation of cells by enhancing FGF-2 signaling^18^, while knockdown of GPC1 in U251 glioma cells reduced cellular growth and proliferation^19^. Furthermore, high expression of GPC1 has been suggested to predict poor prognosis of glioblastoma in a study examining the expression of GPC1 in 53 patients^20^. However, in another study with 31 cases, analysis showed no association between GPC1 expression and any clinical parameters of glioblastoma patients, including overall survival (OS) and tumor grade^21^. Collectively, these results suggest that GPC1 alone cannot act as a strong and stable prognostic indicator for glioma, although GPC1 was implicated in the proliferation of glioma cells. These findings suggested that clarifying the complicated crosstalk of GPC1 with its upstream regulator and combining their expression as a “combination biomarker” will improve the prognostic evaluation for glioma patients. Currently, KRAS and EVI1, two important oncoproteins, have been reported to positively regulate GPC1 expression in pancreatic cell lines^22^. However, the upstream regulator of GPC1 implicated in the proliferation of glioma cells is not fully understood.

Annexin A2 (ANXA2), a calcium-dependent phospholipid-binding protein^23^, is highly expressed in gliomas and positively correlated with malignancy^24,25^. ANXA2 promotes tumor proliferation by upregulating several oncogenes, such as β-catenin, cyclin D1, oncostatin M receptor (OSMR), and c-Myc^26–29^. Nevertheless, whether GPC1, the key regulator of glioma cellular proliferation, is regulated by ANXA2 has not yet been studied. In the present study, we found that ANXA2 increased the expression of GPC1 via c-Myc and that the upregulated GPC1 further promoted the c-Myc level, forming a positive feedback loop, which eventually led to enhanced proliferation of glioma cells. Finally, we utilized a tissue microarray (TMA) and immunohistochemistry to demonstrate that combined indexes of ANXA2 and GPC1 could improve the evaluation of prognosis in glioma patients.

## Materials and methods

### Antibodies and reagents

Anti-β-actin (20536-1-AP) and anti-Glypican 1 (16700-1-AP) antibodies were purchased from Proteintech Group (Rosemont, USA). Anti-Annexin A2 (8235) antibody was purchased from Cell Signaling Technology (MA, USA). Anti-Ki67 (ab16667), anti-PCNA (ab92552), and anti-c-Myc (ab32072) antibodies were purchased from Abcam (Cambridge, UK). The specific c-Myc inhibitor 10058-F4 was purchased from Med Chem Express (NJ, USA).

### Cell culture and tissue specimens

The U118 cell line (HTB-15^™^) was obtained from the ATCC and cultured in Dulbecco’s modified Eagle’s medium (DMEM) supplemented with 10% fetal bovine serum (FBS) and 1% penicillin–streptomycin under a 37 °C humidified atmosphere with 5% CO_2_. The authenticity of the U118 cell line was verified by short tandem repeat analysis within the last 3 years, and all experiments were performed with mycoplasma-free cells.

Two independent cohorts involving 90 and 164 glioma patients were enrolled in this study. To investigate the relationship between ANXA2 and GPC1 in glioma tissues, we collected 90 tumor and peritumoral samples for reverse transcription quantitative polymerase chain reaction (RT-qPCR) analysis (Supplementary Table 1, Cohort 1). The samples were consecutively collected from pathologically verified glioma patients undergoing curative resection at the Department of Neurosurgery, Xiangya Hospital, between September 2017 and June 2019. All specimens were obtained during surgery, immediately frozen in liquid nitrogen and stored at −80 °C. This study was approved by the Ethics Committee of Xiangya Hospital, Central South University (Changsha, China; approval number: 20171211148). Written informed consent was obtained from patients before the samples were collected. To evaluate the prognostic role of ANXA2 in combination with GPC1 in glioma, we used a TMA including 164 paraffin-embedded glioma specimens obtained from Shanghai Outdo Biotech Co., Ltd. (Supplementary Table 1, Cohort 2). Demographic and clinical data were obtained from the patients’ medical records. Detailed clinicopathological features are listed in Supplementary Table 1.

Tumor pathology was determined according to the WHO classification of glioma revised in 2016. Time to recurrence (TTR) was defined as the interval between the date of surgery and the date of diagnosis of relapse. OS was defined as the interval between surgery and death or between surgery and the last observation point.

### Lentiviral infection

Lentiviral vector GV248-mediated expression of control shRNA, two shRNA constructs targeting ANXA2 or GPC1, and lentiviral vector GV358-mediated expression of cDNAs of ANXA2 or GPC1 were obtained from Genechem Co., Ltd. (Shanghai, China), and cells were infected using the above lentiviruses with HitransG P (1×, Shanghai Genechem Co., Ltd.). After 48 h of transduction, the cells were collected and subjected to selection with 1 μg/mL puromycin for 5 days. The shRNA sequences targeting ANXA2 were as follows: sh1, CTGTACTATTATATCCAGCAA and sh2, CCTGCTTTCAACTGAATTGTT. The shRNA sequences targeting GPC1 were as follows: sh1, CTATTGCCGAAATGTGCTCAA and sh2, GACACTGTGCAGTGAGAAGAT. The following nontargeting shRNA sequence was used as the negative control: TTCTCCGAACGTGTCACGT.

### Cell proliferation assays

Cell proliferation was examined using the Cell Counting Kit-8 (CCK8) assay (Japan Dojindo Laboratories) and an EdU kit (Shanghai KeyGene Biotechnology Co., Ltd.) according to the manufacturer’s instructions. For the colony formation assay, 500 viable cells were plated in 6-well plates and cultured overnight. The medium was replaced with DMEM containing 10% FBS and 1% penicillin–streptomycin every 3 days for approximately 14 days. To visualize and count the colonies, we separately used 4% polyoxymethylene (Beijing Solarbio Biotechnology Co., Ltd., P8430) and 0.5% crystal violet (Beijing Solarbio Biotechnology Co., Ltd., G1061) to fix and stain the colonies.

### Western blotting

Cell and tissue protein extracts were prepared using RIPA lysis buffer containing protease and phosphatase inhibitors. Lysates were centrifuged at 12,000*g* for 30 min at 4 °C, and the supernatant was collected. The concentration of the supernatant was determined using a bicinchoninic acid protein quantitative kit (Thermo Fisher Scientific Technology Co., Ltd.). Concentration-normalized lysates were mixed with loading buffer and then boiled at 100 °C for 10 min. Proteins were fractionated by sodium dodecyl sulfate polyacrylamide gel electrophoresis (80 V for 0.5 h and 120 V for 1 h) and transferred onto polyvinylidene fluoride membranes (300 mA for 100 min). The membranes were blocked with Quick blocking solution (Shanghai Beyotime Biotechnology Co., Ltd.) for 15 min, followed by overnight incubation at 4 °C with appropriate dilutions of primary antibodies (Anti-β-actin:1:1000; anti-Glypican 1: 1:1000; Anti-Annexin A2:1:1,000; Anti-Ki67: 1:1,000; anti-PCNA: 1:5000; anti-c-Myc: 1:1000) in antibody diluent solution (Shanghai Beyotime Biotechnology Co., Ltd.). After three washes with TBS-T, the membranes were incubated with the appropriate secondary antibodies for 1 h at room temperature. Three washes with TBS-T were performed and visualized using an Enhanced Chemiluminescence (ECL) Kit (Thermo Fisher Scientific Technology Co., Ltd.) according to the manufacturer’s instructions.

### RNA isolation and RT-qPCR

Total RNA was extracted from tissues or cultured cells using TRIzol reagent (Invitrogen, USA) as described in the protocols. After reverse transcription using cDNA reverse transcriptase (Genecopoeia, Rockville, MD) and Oligo(dT) primers, the mRNA levels of ANXA2, GPC1, and β-actin were analyzed by RT-qPCR using SYBR Green dye (Genecopoeia, Rockville, MD) and Quant Studio 5 RT-qPCR instrument (Applied Biosystems, USA). The mRNAs for ANXA2 and GPC1 were amplified and quantified with the primers listed below. The synthetic oligonucleotide primer sequences for AnnexinA2, Glypican1 and β-actin were as follows: h-AnnexinA2 5′-GATCAGAATCATGGTCTCCCG-3′ (upstream) and 5′-GCCCTTAGTGTCTTGCTGGAT-3′ (downstream); h-Glypican1 5′-TGACTATTGCCGAAATGTGCT-3′ (upstream) and 5′-TCCTGGAGGGCGTTGATG-3′ (downstream); h-β-actin 5′-TAGTTGCGTTACACCCTTTCTTG-3′ (upstream) and 5′-TCACCTTCACCGTTCCAGTTT-3′ (downstream). Amplification conditions were set as follows: predenaturation at 95 °C for 15 min, 40 cycles of denaturation at 95 °C for 15 s, annealing at 60 °C for 30 s, and extension at 72 °C for 60 s. AnnexinA2 and Glypican1 mRNA levels were normalized to that of β-actin.

### Immunohistochemistry analysis

The TMA slides were heated at 65 °C for 30 min, followed by paraffin removal with xylene and subsequent rehydration with ethanol. Antigen retrieval was performed in a chamber containing citrate buffer (pH 6.0) for 20 min and maintained at a sub-boiling temperature. The samples were blocked with 10% goat serum for 1 h at room temperature. Two hundred microliters of primary antibody was added to each microarray slide and incubated overnight at 4 °C (ANXA2, 1:1500; GPC1, 1:50). The signal was developed with 3,3′-diaminobenzidine (DAB), and the slides were counterstained in hematoxylin. The immunostaining analysis of ANXA2 and GPC1 protein expression was performed based on these TMAs. For Pearson correlation analysis, quantification of ANXA2 and GPC1 expression levels was evaluated by Image J software. For prognostic analysis, the immunohistochemistry staining results were converted to an immunoreactive score (IRS). Immunostaining intensity was rated as 0 (negative), 1 (weak), 2 (moderate), and 3 (intense). The extent of positive tumor cells was graded as 0 (negative), 1 (≤10%), 2 (11–50%), 3 (51–80%), and 4 (81–100%). The intensity and extent were multiplied for each score. The results were analyzed using a double-blind method. Five high-power fields (×400) were selected at random, and two experienced pathologists independently evaluated the scores. Specimens of 164 patients were applied to optimize a cutoff of IRS ranging from 0 to 12 by Kaplan–Meier analysis with log-rank test. A score of 8 was set as the cutoff point that best dichotomized patients into high-risk and low-risk groups.

### Statistical analyses

The statistical analyses were performed using SPSS version 17.0 (SPSS statistical software). Correlations between two parameters were performed using Pearson/Spearman correlation analysis. The cumulative recurrence and survival probability were determined using the Kaplan–Meier method, and differences were evaluated using the log-rank test. Cox univariate and multivariate proportional hazards regression analysis was used to determine the independent prognostic factors that influence recurrence and survival. Quantitative data were representative of at least three independent replicates. Quantitative data were compared using two-tailed independent *t* test (between two groups) or one-way ANOVA (among three or more groups). Categorical data were analyzed by the chi-square test or Fisher’s exact test. Sample sizes of all experiments were predetermined by calculations derived from our experience, based on the ability to achieve an overall significance level of *p* = 0.05 and 80% power (*n* = 164/90/6 for each experimental group of the human study and *n* = 3/5 for each experimental group of other in vitro experiments). All values were presented as the mean ± SEM. Differences were deemed statistically significant when *p* < 0.05.

## Results

### ANXA2-induced glioma cell proliferation in a c-Myc-dependent manner

**Fig. 1:**
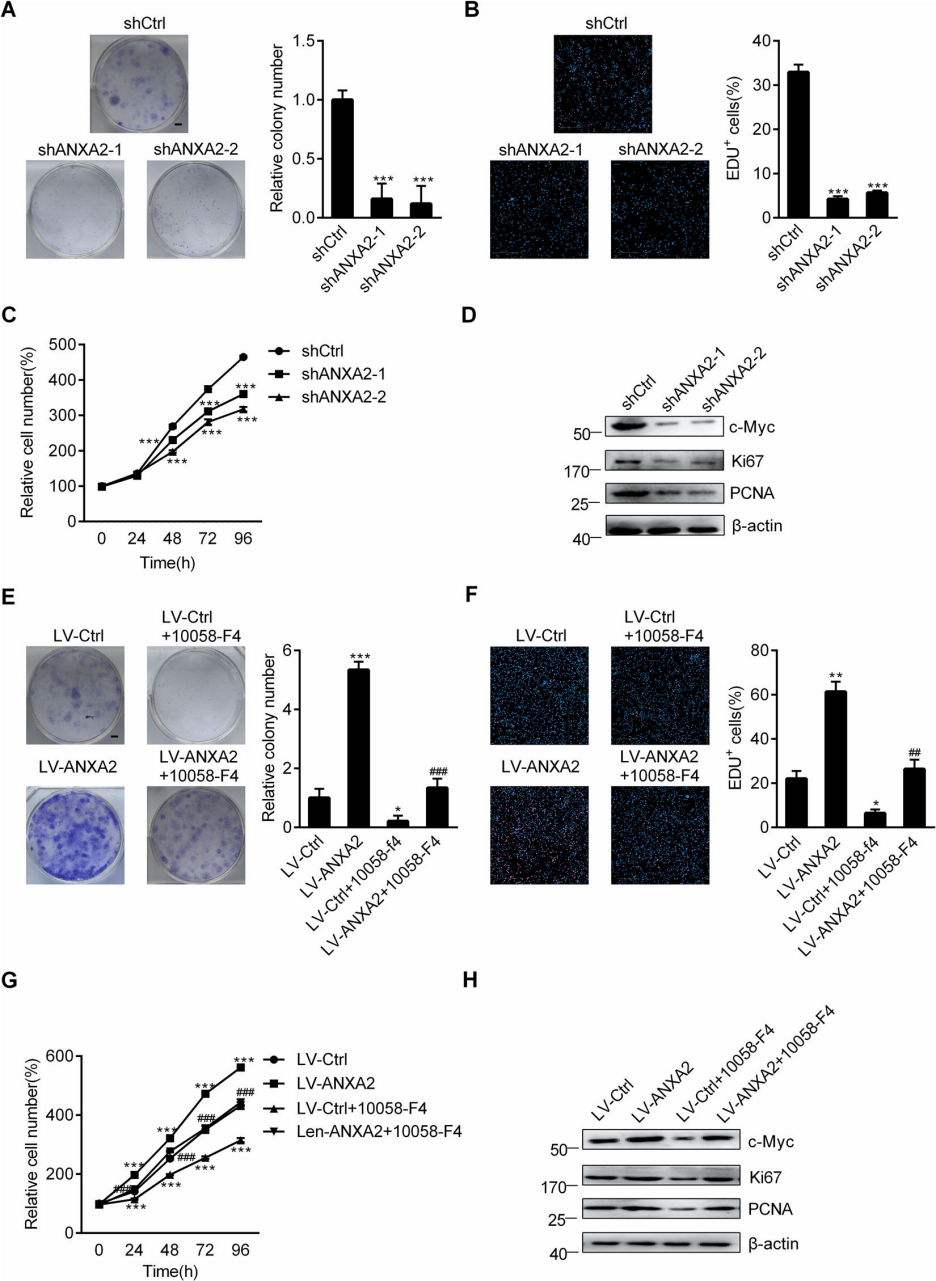
ANXA2 drives glioma cell proliferation in a c-Myc-dependent manner. **A**–**C** Cell proliferation was assessed in stable ANXA2-silenced U118 cells, as shown by colony formation (**A**) (Scale bar = 5 mm), EdU immunofluorescence staining (**B**) (Scale bar = 200 μm) and CCK-8 (**C**) assays. ^∗∗∗^*P* < 0.001 vs. shCtrl. **D** Western blot analysis of c-Myc and proliferation markers (Ki67 and PCNA) in stable ANXA2-silenced U118 cells. **E**–**G** Effects of 24 h of 10058-F4 treatment at 10 μM on the proliferation of U118 cells stably transfected with control or ANXA2 lentiviruses shown by colony formation (**E**) (Scale bar = 5 mm), EdU immunofluorescence staining (**F**) (Scale bar = 200 μm), and CCK-8 (**G**) assays. ^∗^*P* < 0.05; ^∗∗^*P* < 0.01; ^∗∗∗^*P* < 0.001 vs. LV-Ctrl, ^###^*P* < 0.001 vs. LV-ANXA2. **H** Effects of 24 h of 10058-F4 treatment at 10 μM on the protein levels of c-Myc and proliferation markers (Ki67 and PCNA) in U118 cells stably transfected with control or ANXA2 lentiviruses. Error bars represent the standard error of the mean.

To investigate the effect of ANXA2 on glioma cell proliferation, we knocked down ANXA2 in U118 cells using two independent short-hairpin RNAs (shRNAs) (Supplementary Fig. S1A, B). Depletion of ANXA2 significantly inhibited U118 cell proliferation, as shown by colony formation assays, 5-ethynyl-20-deoxyuridine (EdU) staining and CCK-8 assays (Fig. 1A–C). In addition, the expression of Ki67 and PCNA, two proliferation markers, and the oncoprotein c-Myc were all obviously downregulated after ANXA2 was knocked down (Fig. 1D). To further confirm the role of ANXA2 in proliferation, we performed overexpression of ANXA2 in U118 cells (Supplementary Fig. S1C, D). As shown in Fig. 1E–G, the ectopic expression of ANXA2 led to dramatic increases in the number of cell colonies, proportion of EdU-positive cells, and proliferation rate. The levels of Ki67, PCNA, and c-Myc were correspondingly enhanced. However, all these increases could be effectively reversed by a specific inhibitor of c-Myc, 10058-F4 (Fig. 1E–H), indicating that c-Myc is a key modulator in ANXA2-induced glioma cell proliferation.

### A GPC1/c-Myc positive feedback loop was formed in ANXA2-induced glioma cell proliferation

**Fig. 2:**
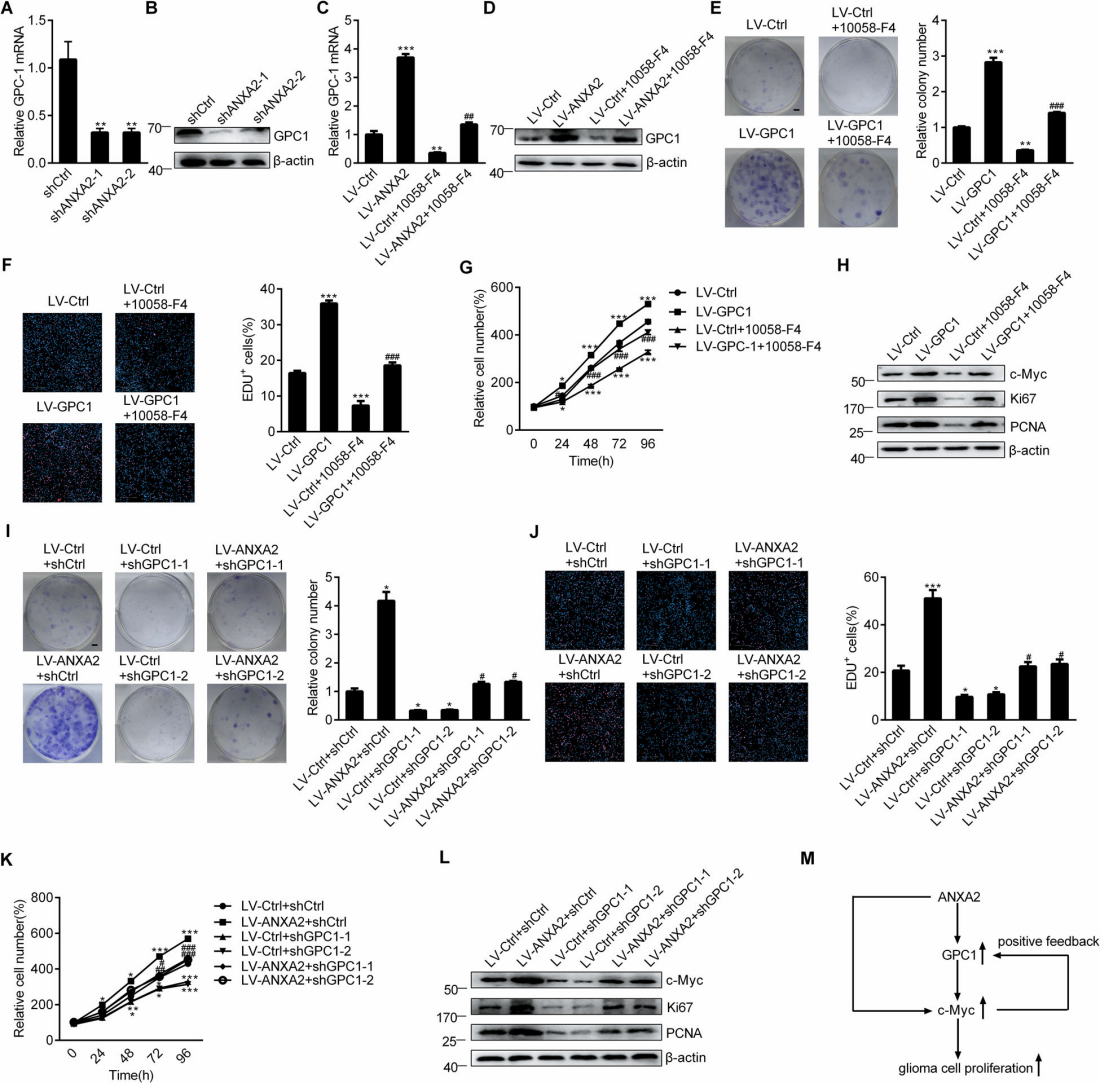
A GPC1/c-Myc positive feedback loop was formed in ANXA2-induced glioma cell proliferation. **A**, **B** The mRNA (**A**) and protein (**B**) levels of GPC1 in stable ANXA2-silenced U118 cells. ^∗∗^*P* < 0.01 vs. shCtrl. **C**, **D** Effects of 24 h of 10058-F4 treatment at 10 μM on the mRNA (**C**) and protein (**D**) levels of GPC1 in U118 cells stably transfected with control or GPC1 lentiviruses. **E**–**G** Effects of 24 h of 10058-F4 treatment at 10 μM on the proliferation of U118 cells stably transfected with control or GPC1 lentiviruses shown by colony formation (**E**) (Scale bar = 5 mm), EdU immunofluorescence staining (**F**) (Scale bar = 200 μm) and CCK-8 (**G**) assays. ^∗^*P* < 0.05; ^∗∗^*P* < 0.01; ^∗∗∗^*P* < 0.001 vs. LV-Ctrl, ^#^*P* < 0.05; ^##^*P* < 0.01; ^###^*P* < 0.001 vs. LV-GPC1. **H** Effects of 24 h of 10058-F4 treatment at 10 μM on the protein levels of c-Myc and proliferation markers (Ki67 and PCNA) in U118 cells stably transfected with control or GPC1 lentiviruses. **I**–**K** U118 cells stably overexpressing ANXA2 were infected with shCtrl or shGPC1 lentiviruses for 72 h or 24–96 h, and cell proliferation was assessed and shown by colony formation (**I**) (Scale bar = 5 mm), EdU immunofluorescence staining (**J**) (Scale bar = 200 μm), and CCK-8 (**K**) assays. ^∗^*P* < 0.05; ^∗∗^*P* < 0.01; ^∗∗∗^*P* < 0.001 vs. LV-Ctrl + shCtrl, ^#^*P* < 0.05; ^##^*P* < 0.01; ^###^*P* < 0.001 vs. LV-ANXA2 + shCtrl. **L** U118 cells stably overexpressing ANXA2 were infected with shCtrl or shGPC1 lentiviruses for 72 h, and western blot analysis of c-Myc and proliferation markers (Ki67 and PCNA) was performed. **M** Schematic summarizing the results demonstrating the GPC1/c-Myc positive feedback loop in ANXA2-induced glioma cell proliferation. Error bars represent the standard error of the mean.

The mRNA and protein levels of GPC1 were confirmed to be decreased in the ANXA2-depleted U118 cells (Fig. 2A, B) and increased in the ANXA2-overexpressing U118 cells compared with the control cells (Fig. 2C, D). The c-Myc inhibitor 10058-F4 significantly inhibited the ANXA2-enhanced GPC1 levels (Fig. 2C, D), indicating that ANXA2 upregulated GPC1 expression is via c-Myc signaling. Then, GPC1 overexpression was performed in glioma cells by infecting them with GPC1-coding lentivirus (Supplementary Fig. S2A, B) to demonstrate the effect of GPC1 on proliferation. Similar to ANXA2, GPC1 overexpression resulted in obvious increases in the number of cell colonies, proportion of EdU-positive cells, proliferative rate, and Ki67, PCNA, and c-Myc levels, which could all be suppressed by the c-Myc inhibitor 10058-F4 (Fig. 2E–H). These data suggest that GPC1, as a downstream target of ANXA2, mediated the proliferation-inducing effect of ANXA2 via c-Myc signaling. To further confirm the contribution of GPC1, we employed two independent shRNAs to knock down GPC1 (Supplementary Fig. S2C, D). As shown in Fig. 2I–L, the knockdown of GPC1 significantly reversed the ANXA2-mediated proliferation of glioma cells and the upregulation of c-Myc. All these results indicated that ANXA2 increased the expression of GPC1 via c-Myc and that the upregulated GPC1 further promoted the c-Myc level, forming a positive feedback loop, which eventually led to enhanced proliferation of glioma cells. The GPC1/c-Myc positive feedback loop initiated by ANXA2 overexpression is shown in Fig. 2M.

### The upregulation of ANXA2 and GPC1 coincided in human glioma

**Fig. 3:**
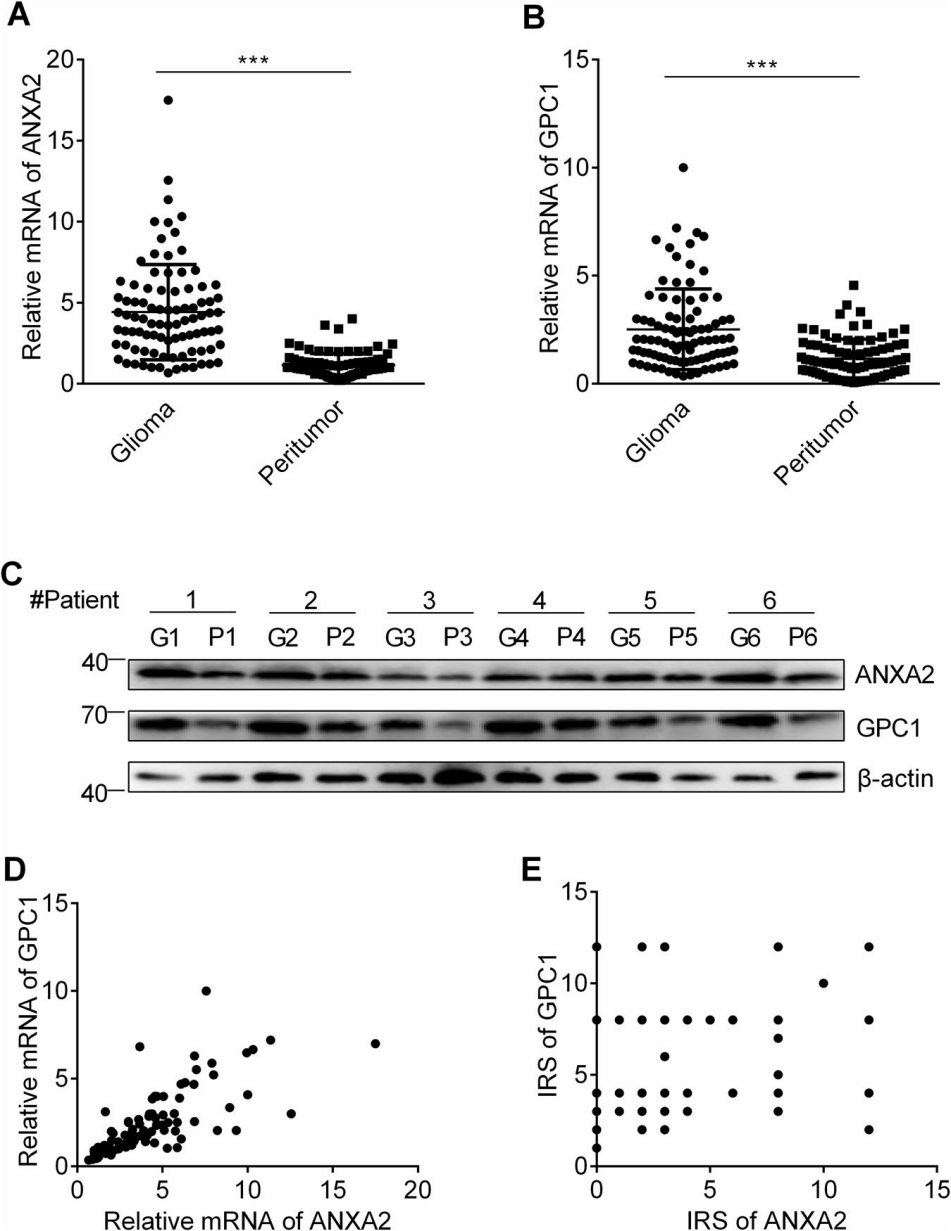
ANXA2 is upregulated in glioma tissues and coincides with upregulation of GPC1 expression. **A**, **B** Relative ANXA2 (**A**) and GPC1 (**B**) mRNA levels among glioma and peritumoral tissues (*n* = 90, ^***^P < 0.001 vs. glioma). **C** The expression of ANXA2 and GPC1 in the glioma (G) and peritumoral (P) tissues (*n* = 6). **D** Positive correlation between ANXA2 and GPC1 in the glioma samples (*n* = 90, *r* = 0.718, *P* < 0.001). **E** Positive correlation between ANXA2 and GPC1 protein levels in the glioma samples (*n* = 164, *r* = 0.23, *P* < 0.01).

The ANXA2 and GPC1 mRNA expression of 90 paired glioma samples in Cohort 1 was measured by qRT-PCR analysis. Both the ANXA2 and GPC1 mRNA levels were significantly overexpressed in the tumor tissues compared with the corresponding peritumoral tissues (*P* < 0.001; Fig. 3A, B). The overexpression of both ANXA2 and GPC1 in tumors was reconfirmed by western blot analyses of 6 paired glioma samples selected from the 90 glioma cases (Fig. 3C). Then, the relationship between ANXA2 and GPC1 was investigated by scatter plot analysis, revealing a significant positive correlation between the ANXA2 and GPC1 mRNA levels in the glioma samples (Pearson’s correlation, *n* = 90, *r* = 0.878, *P* < 0.001; Fig. 3D). Similar results were observed at the protein level in another cohort of patients (*n* = 164, *r* = 0.23, *P* < 0.01; Fig. 3E) by immunohistochemistry.

### Association of ANXA2 and GPC1 expression with the clinicopathological characteristics of glioma

**Fig. 4:**
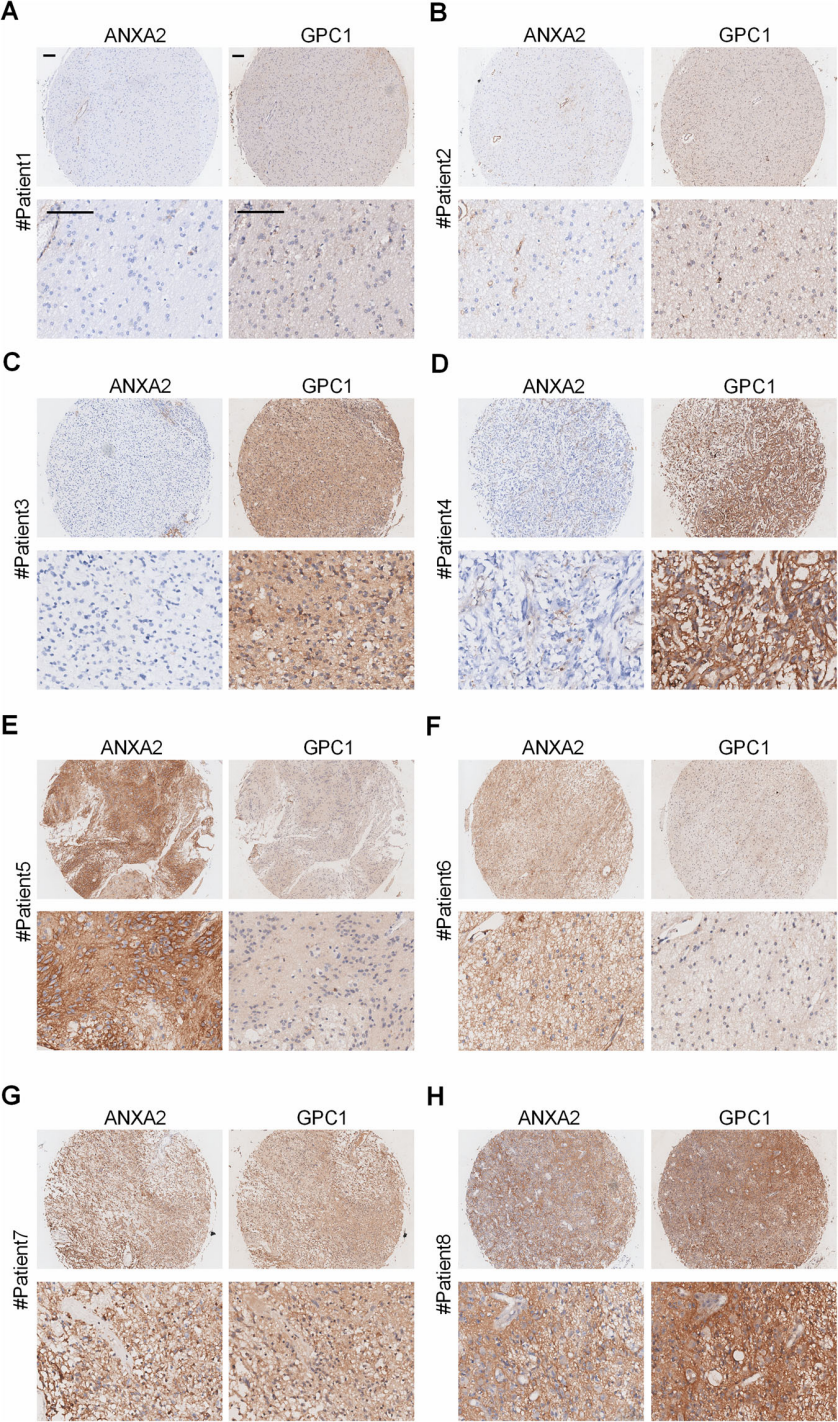
Expression patterns of ANXA2 and GPC1 in glioma samples. **A**–**H** Representative glioma samples show that the expression of ANXA2 (left panel) and GPC1 (right panel) is diverse. **A**, **B** Low expression of both ANXA2 and GPC1. **C**, **D** Low expression of ANXA2 and high expression of GPC1. **E**, **F** High expression of ANXA2 and low expression of GPC1. **G**, **H** High expression of both ANXA2 and GPC1. Scale bar = 50 μm.

The expression levels of ANXA2 and GPC1 were investigated by immunohistochemical staining in a TMA composed of primary tumors from 164 glioma patients in Cohort 2 (Supplementary Table 1 and Table 1). ANXA2 and GPC1 proteins were both primarily located on the cell membrane and in the cytoplasm of tumor cells (Fig. 4A–H). According to the immunohistochemistry data, we classified the glioma patients into four groups by the protein levels of ANXA2 and GPC1: (1) patients with both low ANXA2 and GPC1 levels (ANXA2^low^/GPC1^low^ patients) ((*n* = 69); (2) those with low ANXA2 and high GPC1 levels (ANXA2^low^/GPC1^high^ patients) (*n* = 54); (3) those with high ANXA2 and low GPC1 levels (ANXA2^high^/GPC1^low^ patients) (*n* = 18); and (4) those with both high ANXA2 and GPC1 levels (ANXA2^high^/GPC1^high^ patients) (*n* = 23) (Fig. 4A–H). Then, an association analysis was performed between ANXA2 and GPC1 expression and the clinicopathological features of glioma patients. Person’s chi-square test indicated that the combined expression of ANXA2 and GPC1 was dramatically associated with age (*P* < 0.001), WHO grade (*P* = 0.005), recurrence (*P* < 0.001), and survival status (*P* < 0.001) but not with gender or tumor location (Table 1).

**Table 1:** Correlation between the factors and clinicopathologic characteristics in glioma (*n* = 164).

Clinicopathological indexes	Combination of ANXA2 and GPC1^a^				
	I	II	III	IV	*P*
Age					
≤50	56	39	13	7	<0.001
>50	13	15	5	16	
Gender					
Male	43	33	12	17	0.725
Female	26	21	6	6	
Location					
Frontal	23	14	4	6	0.567
Temporal	19	19	5	10	
Parietal	4	5	0	2	
Occipital	2	3	2	2	
Others	21	13	7	3	
WHO grade					
I + II	49	29	9	7	0.005
III + IV	20	25	9	16	
Recurrence					
Absence	46	18	12	0	<0.001
Presence	23	36	6	23	
Status					
Live	52	37	13	5	<0.001
Death	17	17	5	18	

Chi-square test or Fisher’s exact test for all the analyses.

*P* values less than 0.05 were considered statistically significant.

^a^I, ANXA2^low^/GPC1^low^; II, ANXA2^low^/GPC1^high^; III, ANXA2^high^/GPC1^low^; IV, ANXA2^high^/GPC1^high^.

### High expression of both ANXA2 and GPC1 predicted poor prognosis in glioma patients

**Fig. 5:**
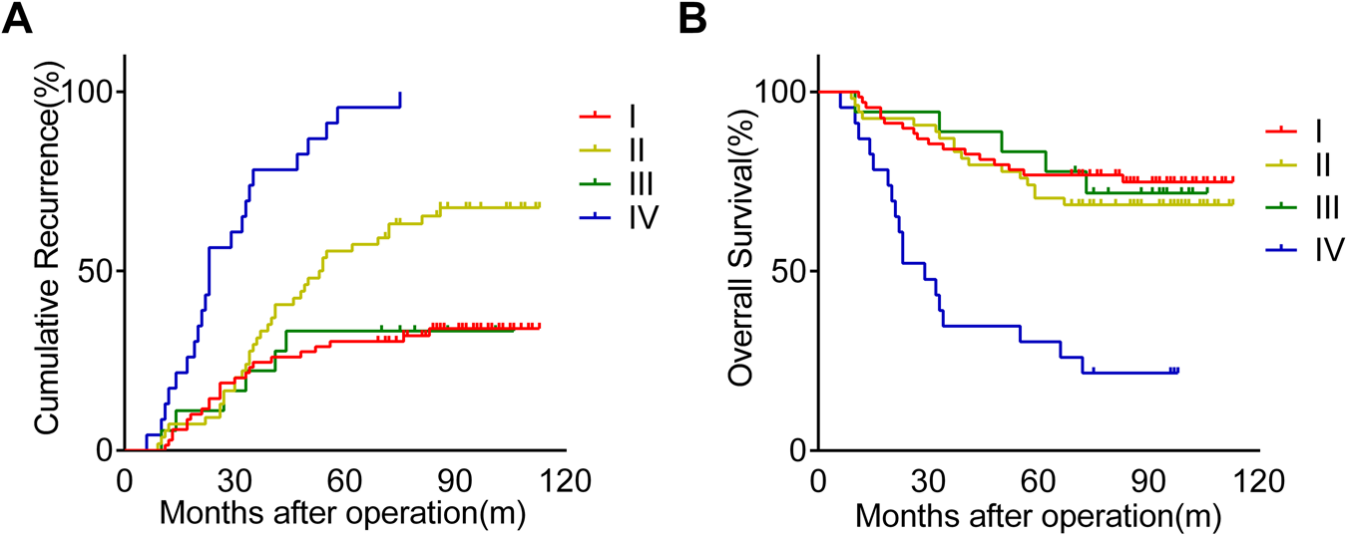
Kaplan–Meier curves of recurrence and survival among glioma patients. **A**, **B** Glioma patients with both high ANXA2 and GPC1 expression had the poorest prognoses as measured by cumulative recurrence and overall survival. **A** Kaplan–Meier analyses of cumulative recurrence in glioma patients. *P* (I vs. IV) < 0.001; *P* (II vs. IV) < 0.001; *P* (III vs. IV) < 0.001; *P* (I vs. II) < 0.01; *P* (III vs. II) < 0.05. **B** Kaplan–Meier analyses of overall survival in glioma patients. *P* (I vs. IV) < 0.001; *P* (II vs. IV) < 0.001; *P* (III vs. IV) < 0.01. I, ANXA2^low^/GPC1^low^; II, ANXA2^low^/GPC1^high^ III, ANXA2^high^/GPC1^low^; and IV, ANXA2^high^/GPC1^high^.

By the last follow-up, 53.8% (92/164) of the patients in Cohort 2 had suffered from glioma recurrence, and 33.3% (57/164) had died. The 1-, 3-, and 5-year cumulative recurrence rates were 6.7%, 34.1% and 48.2%, respectively. The 1-, 3-, and 5-year OS rates in the whole cohort were 93.9%, 78.7%, and 68.9%, respectively. When ANXA2 and GPC1 expression was taken into consideration, we found that the 1-, 3-, and 5-year cumulative recurrence rates in the ANXA2^high^/GPC1^high^ patients were 17.4%, 78.3%, and 95.7%, respectively, and were significantly higher than the 1-, 3-, and 5-year cumulative recurrence rates for the ANXA2^low^/GPC1^high^ (7.4%, 31.5%, and 55.6%, respectively), ANXA2^high^/GPC1^low^ (5.6%, 22.2%, and 33.3%, respectively) and ANXA2^low^/GPC1^low^ (2.9%, 24.6%, and 30.4%, respectively) patients (Fig. 5A). When compared with the ANXA2^low^/GPC1^low^ or ANXA2^high^/GPC1^low^ group, the ANXA2^low^/GPC1^high^ group presented higher 1-, 3-, and 5-year cumulative recurrence rates (Fig. 5A). No significance was found in the 1-, 3-, and 5-year cumulative recurrence rates between the ANXA2^low^/GPC1^low^ and ANXA2^high^/GPC1^low^ groups (Fig. 5A). The 1-, 3-, and 5-year OS rates in the ANXA2^high^/GPC1^high^ patients were 87%, 34.8%, and 26.1%, respectively, which were significantly lower than those for the ANXA2^low^/GPC1^high^ (92.6%, 83.3%, and 70.4%, respectively), ANXA2^high^/GPC1^low^ (94.4%, 88.9%, and 77.8%, respectively) and ANXA2^low^/GPC1^low^ (97.1%, 84.1%, and 76.8%, respectively) patients (Fig. 5B). No significance was found in the 1-, 3-, and 5-year OS rates among the ANXA2^low^/GPC1^low^, ANXA2^low^/GPC1^high^, and ANXA2^high^/GPC1^low^ groups, although the ANXA2^low^/GPC1^high^ group tended to have a lower OS rate than the ANXA2^low^/GPC1^low^ and ANXA2^high^/GPC1^low^ groups (Fig. 5B). Furthermore, univariate and multivariate analyses revealed that in addition to WHO grade and age, the combination of ANXA2 and GPC1 (ANXA2/GPC1) was an independent prognostic factor for TTR (*P* < 0.001, HR = 3.575, respectively) and OS (*P* = 0.001, HR = 2.174, respectively) in glioma patients (Table 2).

**Table 2:** Univariate and multivariate analyses of prognostic factors with TTR and OS in glioma (*n* = 164).

Variables	TTR		OS	
	HR (95% CI)	*P*	HR (95% CI)	*P*
*Univariate analysis*^a^				
Age (≤50 vs. å 50)	2.189 (1.428–3.355)	<0.001	2.542 (1.510–4.277)	<0.001
Gender (male vs. female)	0.731 (0.466–1.145)	0.171	0.703 (0.398–1.239)	0.223
Location		0.063		0.019
(Frontal vs. temporal)	1.720 (1.007–2.938)	0.047	1.661 (0.841–3.281)	0.144
(Frontal vs. parietal)	1.175 (0.476–2.898)	0.727	1.284 (0.419–3.940)	0.662
(Frontal vs. occipital)	2.190 (0.934–5.137)	0.072	3.775 (0.760–1.377)	0.005
(Frontal vs. others)	0.876 (0.470–1.634)	0.678	0.283 (1.501–9.494)	0.643
WHO grade (I + II vs. III + IV)	6.348 (3.997–10.082)	<0.001	13.968 (0.760–1.377)	<0.001
Combination of ANXA2 and GPC1^b^		<0.001		<0.001
(I vs. IV)	7.356 (4.043–13.385)	<0.001	5.396 (2.763–10.539)	<0.001
(II vs. IV)	3.162 (1.849–5.404)	<0.001	4.178 (2.140–8.159)	<0.001
(III vs. IV)	7.268 (2.921–18.081)	<0.001	4.987 (1.842–13.501)	0.002
(I vs. III)	1.012 (0.412–2.486)	0.979	1.082 (0.399–2.933)	0.877
(I vs. II)	2.327 (1.376–3.935)	0.002	1.291 (0.659–2.930)	0.456
(III vs. II)	2.299 (0.968–5.461)	0.059	1.194 (0.440–3.235)	0.728
*Multivariate analysis*^a^				
Age (≤50 vs. å 50)	1.716 (1.985–2.716)	0.021	1.891 (1.067–3.353)	0.029
Gender (male vs. female)	NA	NA	NA	NA
Location		0.447		0.058
(Frontal vs. temporal)	1.515 (0.875–2.621)	0.138	1.618 (0.803–3.261)	0.178
(Frontal vs. parietal)	0.946 (0.375–2.386)	0.907	1.469 (0.466–4.631)	0.512
(Frontal vs. occipital)	1.892 (0.781–4.581)	0.158	4.394 (1.658–11.649)	0.003
(Frontal vs. others)	1.361 (0.698–2.653)	0.365	1.461 (0.604–3.529)	0.400
WHO grade (I + II vs. III + IV)	5.703 (3.475–9.358)	<0.001	14.294 (6.523–31.323)	<0.001
Combination of ANXA2 and GPC1^b^		<0.001		0.001
(I vs. IV)	3.575 (1.880–6.799)	<0.001	2.174 (0.760–1.377)	0.045
(II vs. IV)	2.341 (1.340–4.092)	0.003	3.459 (1.685–7.104)	0.001
(III vs. IV)	7.352 (2.833–19.081)	<0.001	5.754 (1.987–16.663)	0.001
(I vs. III)	0.486 (0.190–1.244)	0.133	0.379 (0.132–1.082)	0.070
(I vs. II)	1.527 (0.883–2.640)	0.130	0.629 (0.309–1.281)	0.201
(III vs. II)	3.140 (1.266–7.787)	0.014	1.663 (0.587–4.717)	0.339

*TTR* time to recurrence, *OS* overall survival, *HR* hazard ratio, *CI* confidential interval, *NA* not adopted.

*P* values less than 0.05 were considered statistically significant.

^a^Cox proportional hazards regression.

^b^I, ANXA2^low^/GPC1^low^; II, ANXA2^low^/GPC1^high^; III, ANXA2^high^/GPC1^low^; IV, ANXA2^high^/GPC1^high^.

## Discussion

Rapid and uncontrollable proliferation is one of the main reasons for the high recurrence and death rate of glioma^30–33^. Growing evidence has shown that effective suppression of glioma cell proliferation could improve the outcomes of glioma patients^30,34^. Although some factors that regulate the proliferation of glioma have been reported, more detailed mechanisms remain unclear.

Previous studies have suggested that ANXA2 is a crucial protein involved in the overproliferation of various human malignant tumor cells^26,27,35,36^, including glioma cells^37–39^. Knockdown of ANXA2 inhibited the proliferation of the glioma cell lines U251, U87, and GP1^37,38^, primary glioma cells^28,39^ and glioblastoma stem-like cells^28,39^. In our present study, we demonstrated that ANXA2 depletion significantly decreased the proliferation of human U118 glioma cells. Furthermore, we demonstrated that the overexpression of ANXA2 could obviously increase the proliferation of U118 glioma cells, which express higher levels of endogenous ANXA2 than the normal human astrocyte cell line NHA (data not shown). Similar findings have been reported in U251 glioma cells and primary patient-derived glioblastoma cells^28,37^. Collectively, these studies support the contribution of ANXA2 to promoting the proliferation of glioma cells, and the overexpression of ANXA2 was sufficient to further increase the proliferation of glioma cells, which already expressed high levels of exogenous ANXA2. However, the promotion of cell proliferation has not been observed in ANXA2-overexpressing primary glioblastoma stem-like cells^39^. One possible reason for this discrepancy might be the different properties between glioblastoma stem-like cells and glioma cells^40,41^.

ANXA2 was shown to regulate OSMR expression via STAT3 phosphorylation, resulting in the shift of glioblastoma cells towards a mesenchymal phenotype with a prominent proliferative capability^28^. Chen et al. reported that ANXA2 knockdown led to a reduction in phosphorylated STAT3^(Y705)^ through direct binding with STAT3 and suppression of STAT3-cyclin D1 pathway-mediated cell proliferation, which nevertheless could not be activated by ANXA2 overexpression^37^. In our present data, both ANXA2 knockdown and overexpression influenced the level of c-Myc and then affected cell proliferation in U118 cells. We further confirmed that ANXA2 induced glioma cell proliferation in a c-Myc-dependent manner by using a specific c-Myc inhibitor. ANXA2 directly regulates the level of c-Myc at the post-transcriptional level by binding to c-Myc mRNA^42^. Apart from the direct regulation of ANXA2 on c-Myc, other mechanisms mediating the activation of c-Myc by ANXA2 are still unclear.

GPCs are known to be expressed in several cancers and have attracted attention as possible biomarkers for the progression of various cancers. At present, the roles of GPC1 and GPC3 in controlling the proliferation of glioma cells have been reported; the overexpression of GPC1 in U87 glioma cells enhanced FGF-2-stimulated proliferation of cells by enhancing FGF-2 signaling^18^, whereas knockdown of GPC1 in U251 glioma cells reduced cellular growth and proliferation^19^; GPC3 drove gliomagenesis and initiated brain hyperexcitability^43^. In fact, the expression of GPC1 and GPC3 appears to be highly tissue specific. Of six GPCs, GPC1 is abundantly expressed in the brain^44^. Although GPC3 protein expression was recently detected in human glioma tissues by immunohistochemistry^43^, Pilia previously reported that GPC3 mRNA is not detectable in the brain^45^. In view of the fact that GPC1 is more abundantly expressed in the brain, we focused our study on GPC1 and demonstrated that the proliferation of U118 glioma cells was promoted by GPC1 overexpression but weakened by GPC1 deletion. The data indicated that even without the stimulation of FGF-2, the pro-proliferative effect of GPC1 could be present in U118 glioma cells, which contain high levels of endogenous GPC1 (data not shown). Currently, KRAS and EVI1, two important oncoproteins, have been reported to upregulate GPC1 expression in pancreatic cell lines^22^. To our knowledge, the data reported herein demonstrating that ANXA2 increased the expression of GPC1 at both the mRNA and protein levels are among the first to suggest that the oncoprotein ANXA2 is an upstream regulator of GPC1. In fact, ANXA2, an RNA-binding protein, recognizes a specific sequence 5′-AA(C/G)(A/U)G in the 3′-UTR of its cognate mRNA, thus post-transcriptionally regulating the expression of these specific genes^46,47^. However, the cognate mRNA of GPC1 does not contain the specific sequence 5′-AA(C/G)(A/U)G in the 3′-UTR. Thus, it is possible that ANXA2 regulates the protein synthesis of GPC1 by mechanisms other than a specific sequence-dependent mechanism. Further, our results unequivocally demonstrated that ANXA2 increased GPC1 expression in a c-Myc-dependent manner. Notably, ANXA2-induced GPC1 overexpression, in turn, caused a sustained upregulation of c-Myc, leading to enhanced glioma cell proliferation. The data suggest that ANXA2 induces a positive feedback loop involving GPC1 and c-Myc to amplify the proliferation of glioma cells (Fig. 2M). A similar positive feedback signaling loop of GPC3 and c-Myc has been demonstrated in hepatocellular carcinoma; c-Myc upregulated the expression of GPC3 by binding to its c-Myc-binding sites in the GPC3 promoter, while GPC3 elevated the expression of c-Myc^48^. Despite the significant sequence homology between the GPC1 and GPC3 genes^49^, further studies are needed to determine whether GPC1 and GPC3 share a similar mechanism of crosstalk with c-Myc.

We subsequently studied the expression of ANXA2 and GPC1 in clinical glioma samples. In 90 tumors (Cohort 1), we found that both ANXA2 and GPC1 were more highly expressed at the mRNA level in the glioma samples than in the peritumoral tissues. Similar results were obtained at the protein level in the glioma tissues of 6 patients. Because our in vitro results confirmed that ANXA2 promoted proliferation by regulating GPC1, we speculated that GPC1 overexpression in glioma may be attributed to the dysregulated expression of ANXA2. Intriguingly, a positive relationship between ANXA2 and GPC1 was demonstrated at both the mRNA and protein levels in glioma tissues. Considering the crosstalk between ANXA2 and GPC1, we further determined whether coexpression of ANXA2 and GPC1 could act as a predictor of glioma prognosis. We found that the subgroup of patients presenting with ANXA2^high^/GPC1^high^ were more prone to recurrence and suffered worse survival rates. Conversely, glioma patients who expressed low levels of either ANXA2 or GPC1 had a better prognosis.

In conclusion, ANXA2 promotes glioma cell proliferation by forming a positive feedback loop between GPC1 and c-Myc, indicating a potential and promising target for glioma treatment in future studies. Most importantly, the combination of ANXA2 and its downstream target GPC1 can be used for improved prognostic evaluation in glioma patients.

## Supplementary information

supplemental materials of the manuscript

## Data Availability

The datasets used and/or analyzed during the current study are available from the corresponding author on reasonable request.
