# Supplementary material for: Overexpression of Annexin A2 promotes proliferation by forming a Glypican 1/c-Myc positive feedback loop: prognostic significance in human glioma
Source: Cell Death Dis. 2021 Mar 12;12(3):261. doi: 10.1038/s41419-021-03547-5 (PMC7954792; doi:10.1038/s41419-021-03547-5)

**Supplemental Figure 1**

**
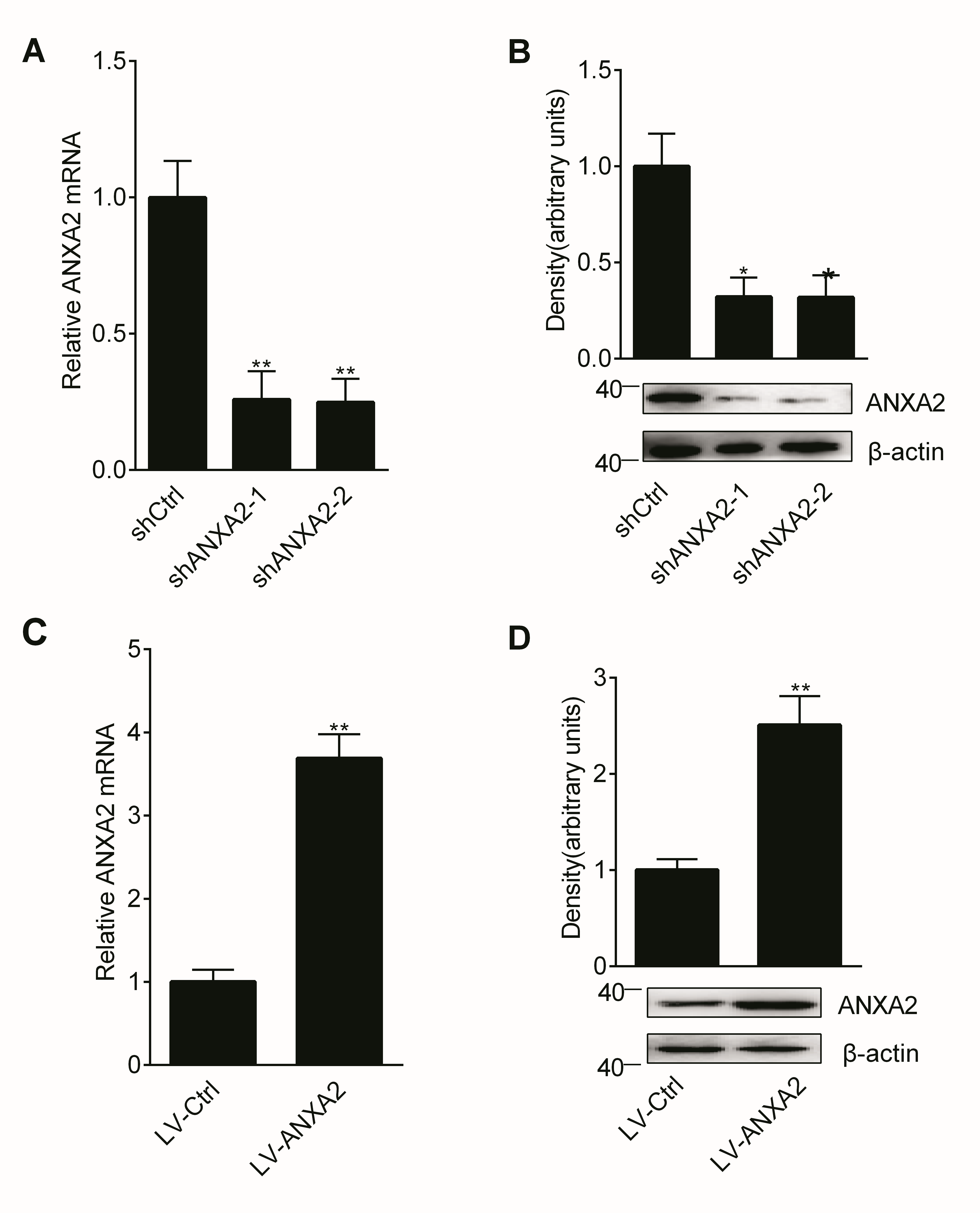
**

**Fig. S1** The knockdown and overexpression of ANXA2. **A-B**, The mRNA (**A**) and protein (**B**) levels of ANXA2 in ANXA2 stably silenced U118 cells. *, *P* < 0.05; **, *P* < 0.01 vs. shCtrl. **C-D**, The mRNA (**C**) and protein (**D**) levels of ANXA2 in U118 cells stably overexpressing ANXA2. **, *P* < 0.01 vs. LV-Ctrl. Error bars represent standard error of mean.

**Supplemental Figure 2**


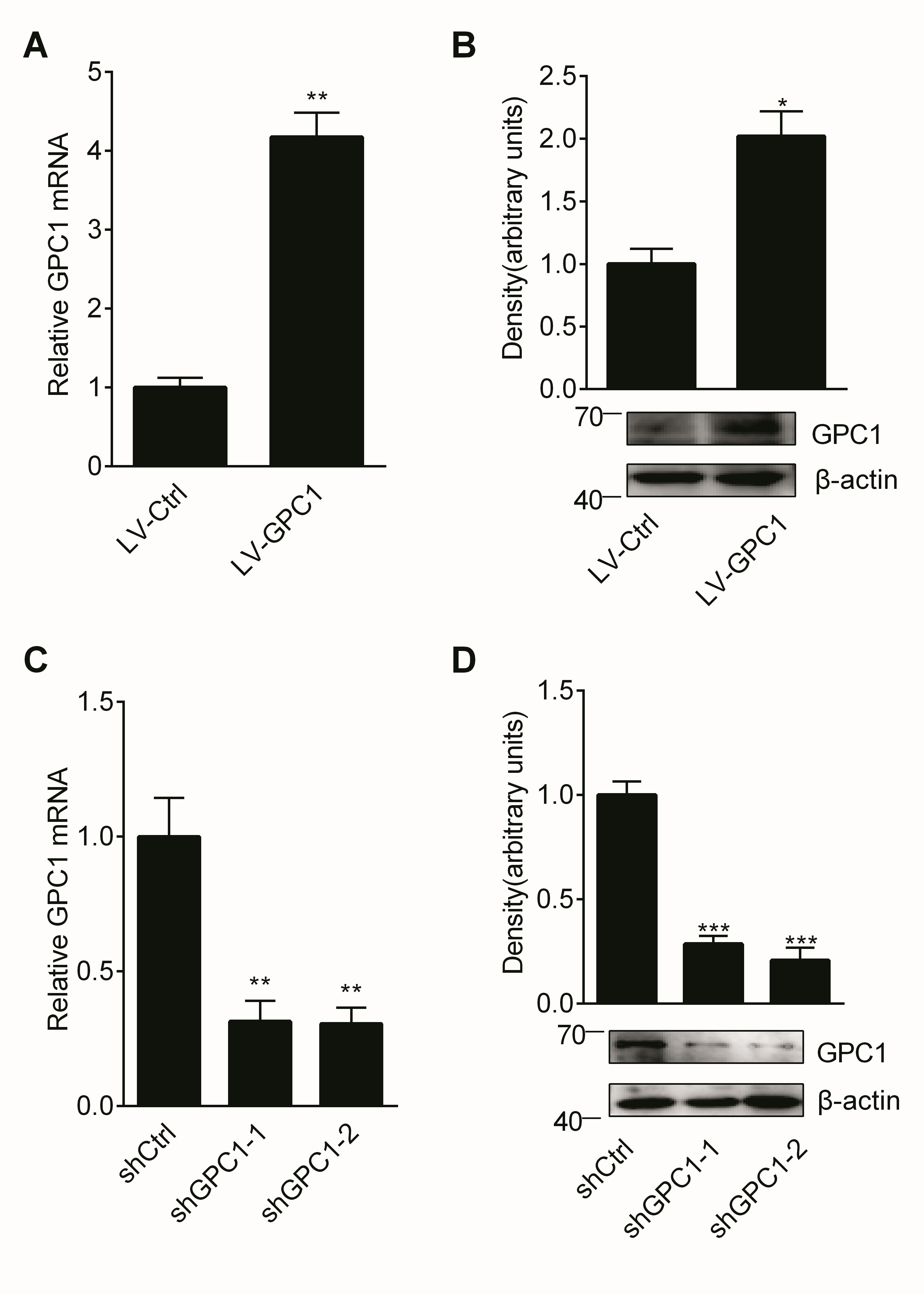


**Fig. S2** The overexpression and knockdown of GPC1. **A-B**, The mRNA (**A**) and protein (**B**) levels of GPC1 in U118 cells stably overexpressing GPC1. *, *P* < 0.05; **, *P* < 0.01 vs. LV-Ctrl. **C-D**, The mRNA (**C**) and protein (**D**) levels of GPC1 in GPC1 stably silenced U118 cells. **, *P* < 0.01; ***, *P* < 0.001 vs. shCtrl. Error bars represent standard error of mean.

**Supplemental Table 1. Clinicopathologic characteristics of glioma patients in two cohorts**

| **Clinicopathological indexes** |  | Cohort 1 (90 cases) |  | Cohort 2 (164 cases) |  |
| --- | --- | --- | --- | --- | --- |
|  |  | N | % | N | % |
| Age(year)  Gender  Location  WHO Grade  Recurrence  Status | ≤50  >50  Male  Female  Frontal  Temporal  Parietal  Occipital  Others  I+ II  III+ IV  Absence  Presence  Live  Death | 51  39  48  42  31  27  13  7  2  14  76  /  /  /  / | 56.7  43.3  53.3  46.7  17  83  98.9  1.1  31.9  15.6  84.4  /  /  /  / | 115  49  105  59  47  53  11  9  44  94  70  76  88  107  57 | 70.1  29.9  64.0  36.0  28.7  32.3  6.7  5.5  26.8  57.3  42.7  46.3  53.7  65.2  34.8 |

**Supplemental Western Blotting Figures**

Fig.1D


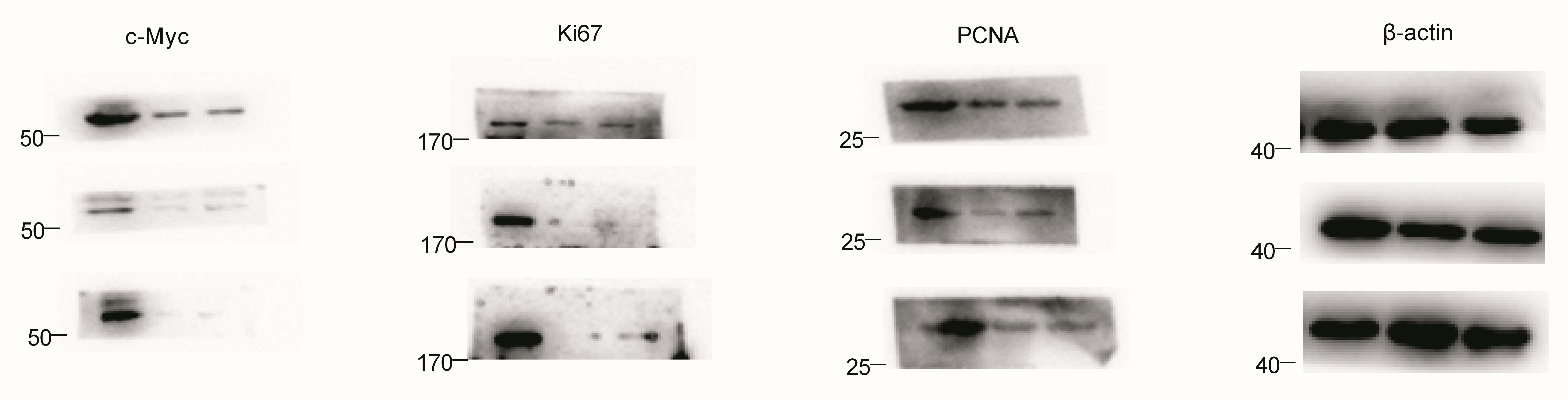


Fig.1H


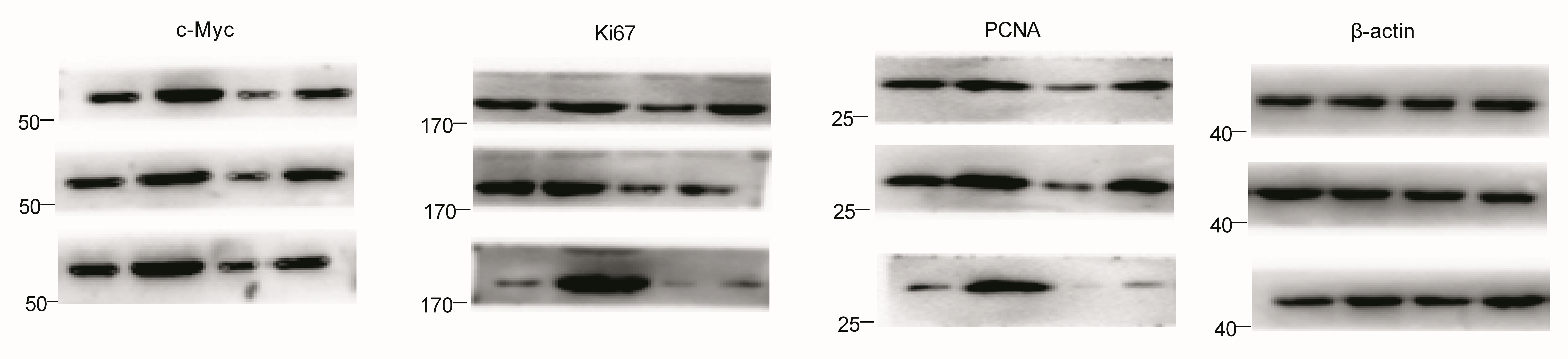


Fig.2B


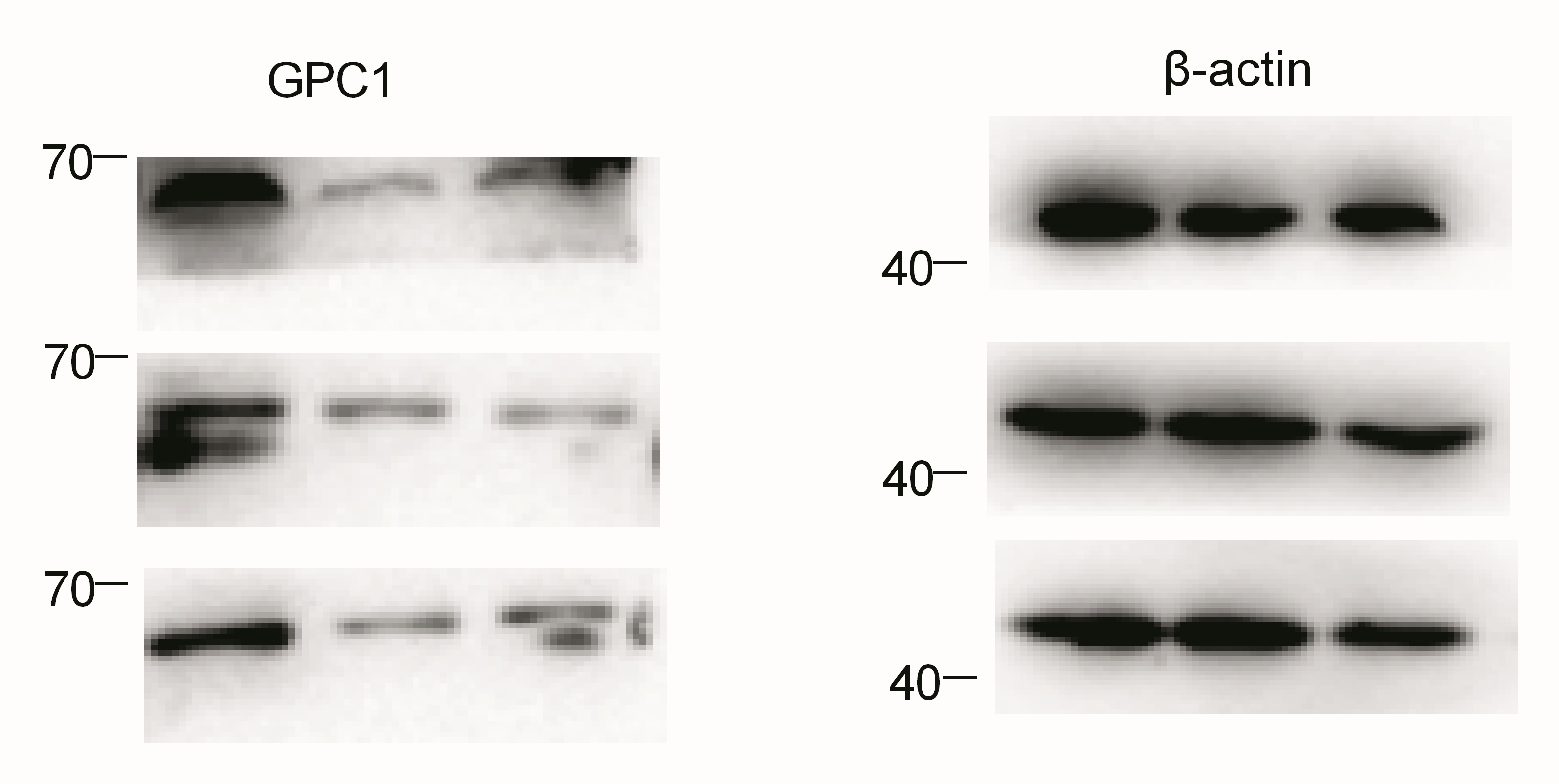


Fig.2D


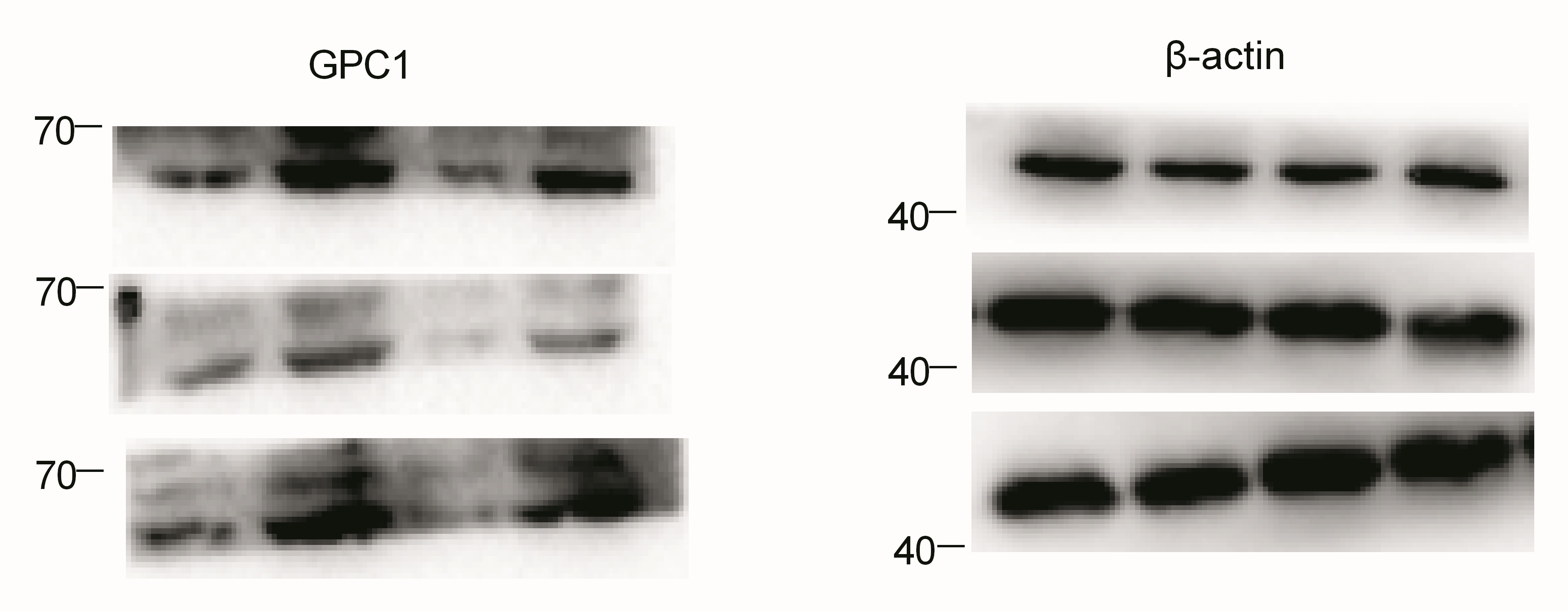


Fig.2H


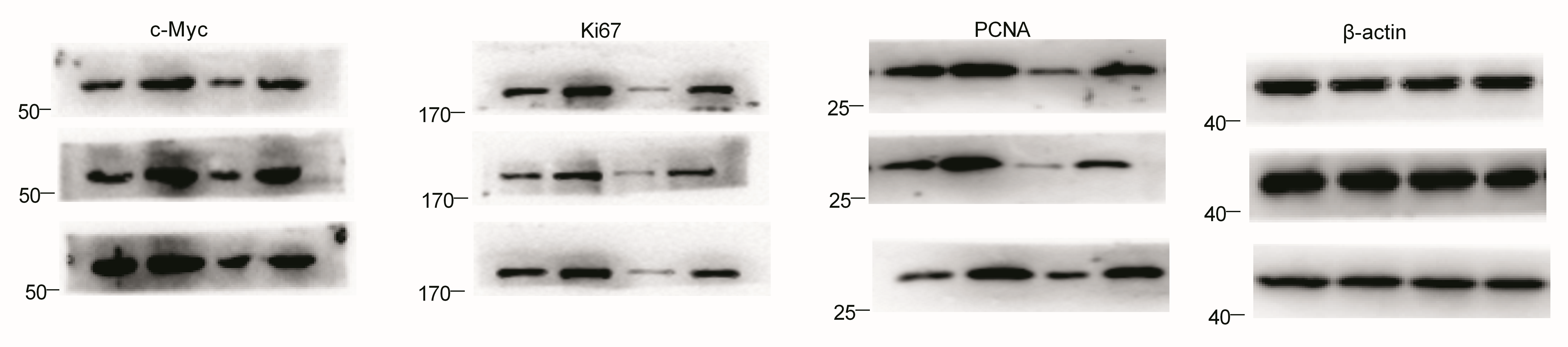


Fig.2L


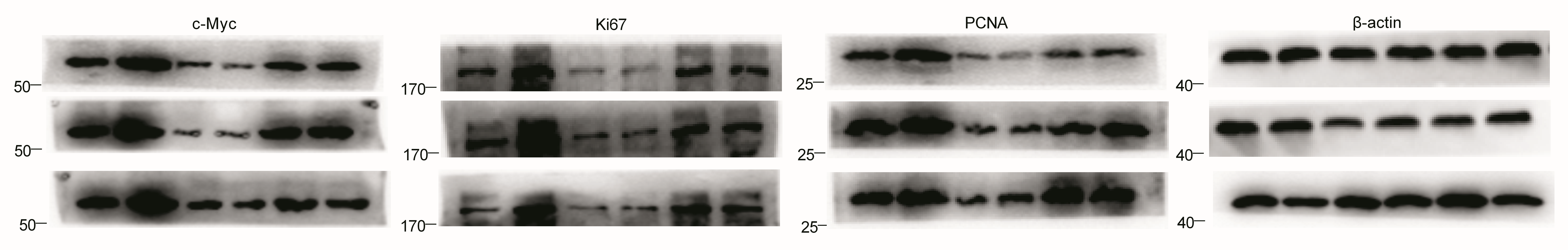


Fig.3C


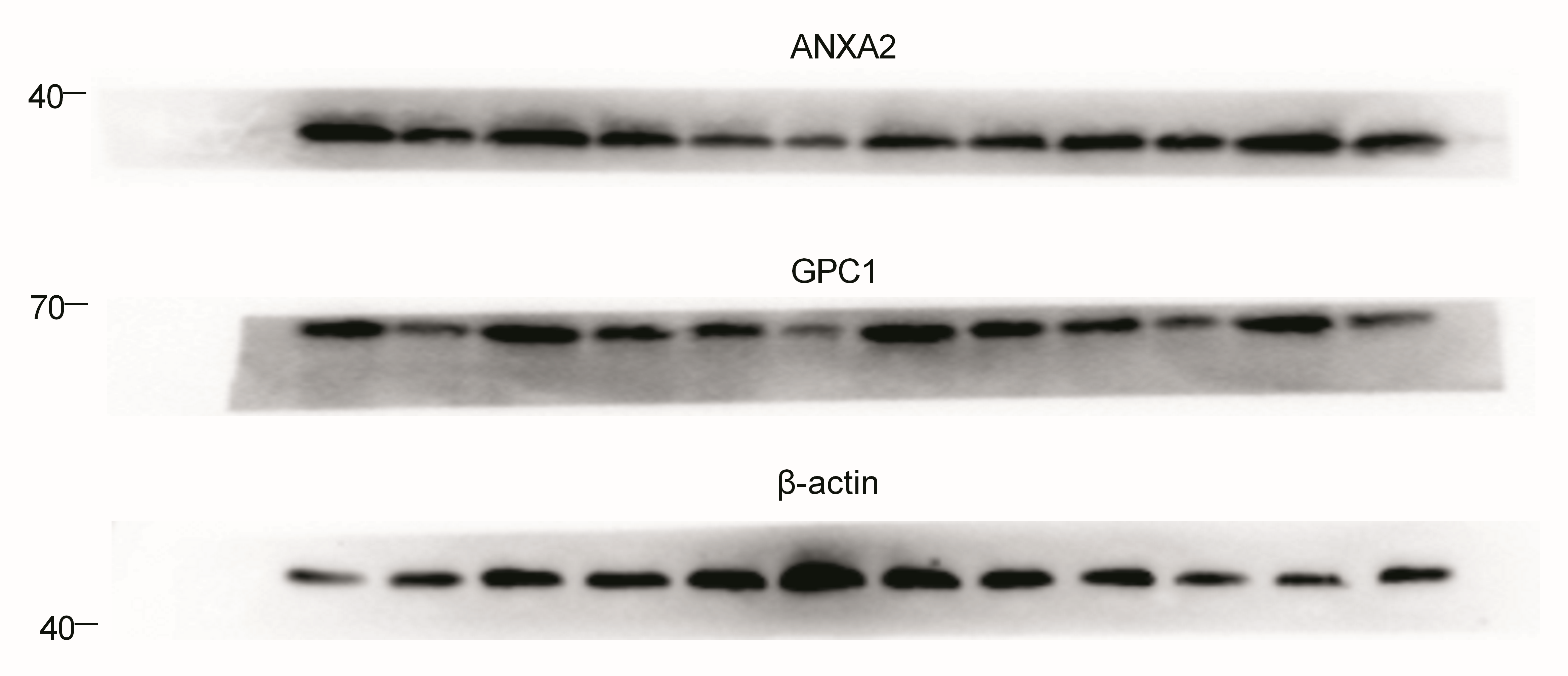


Fig.S1B


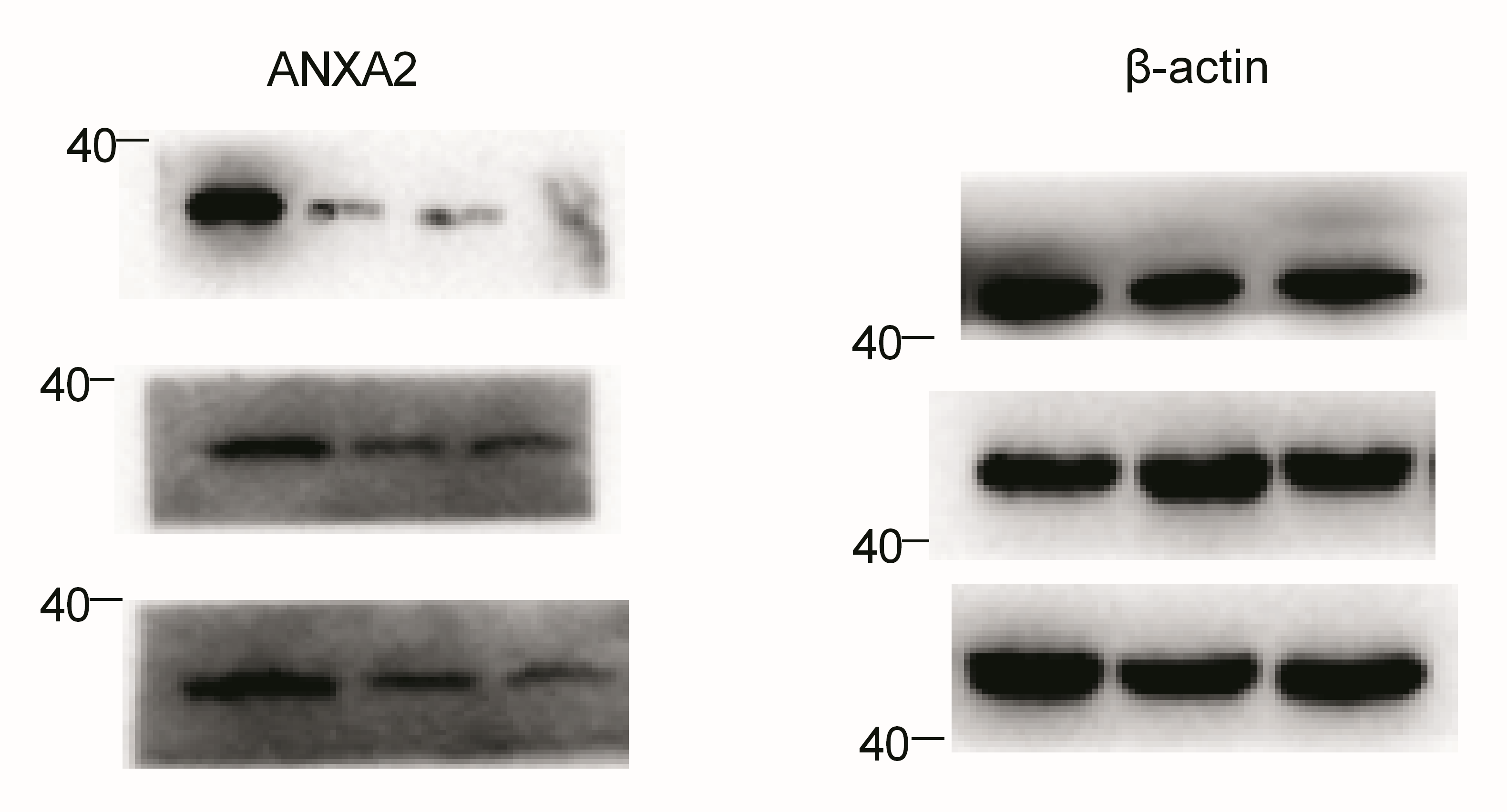


Fig.S1D


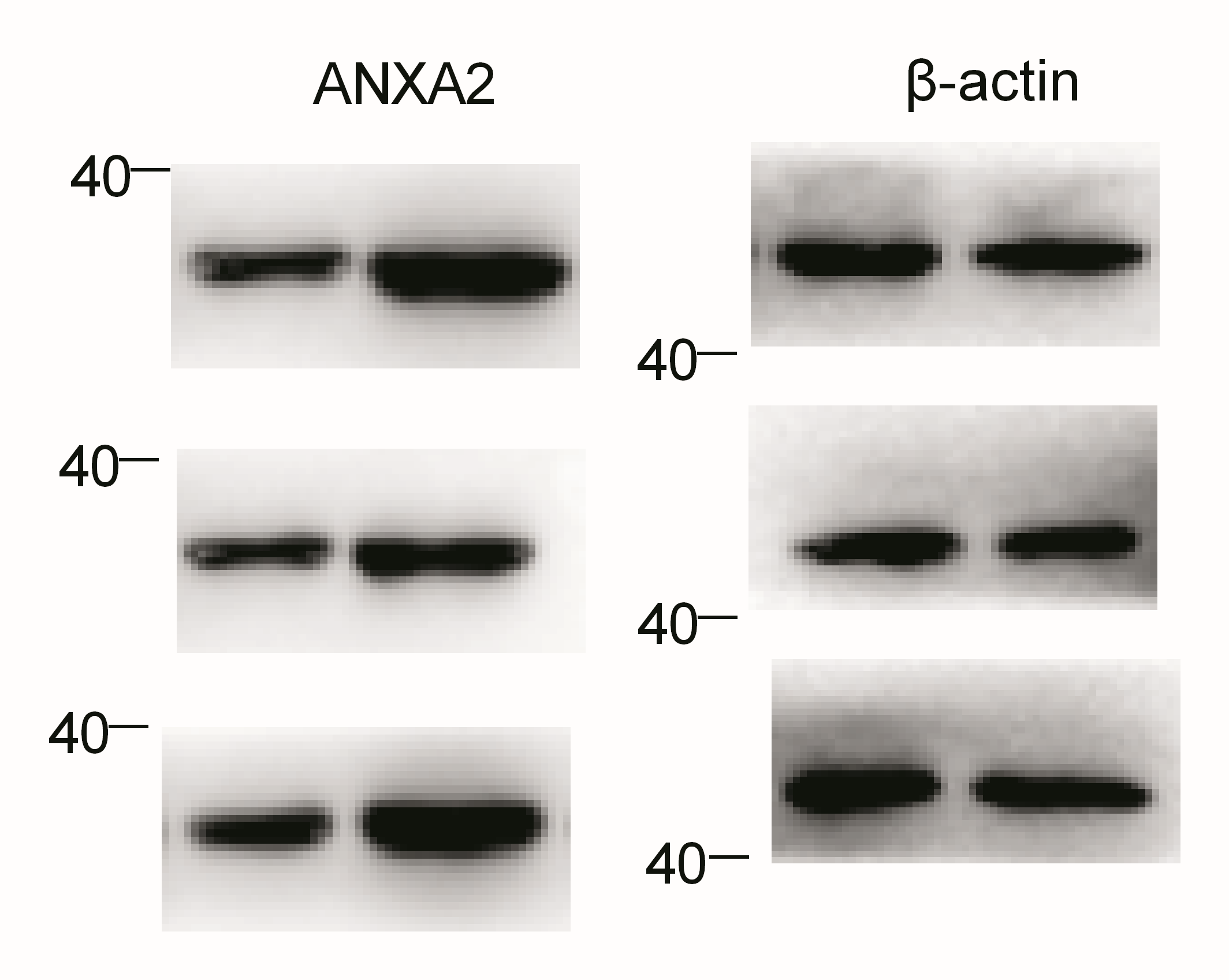


Fig.S2B


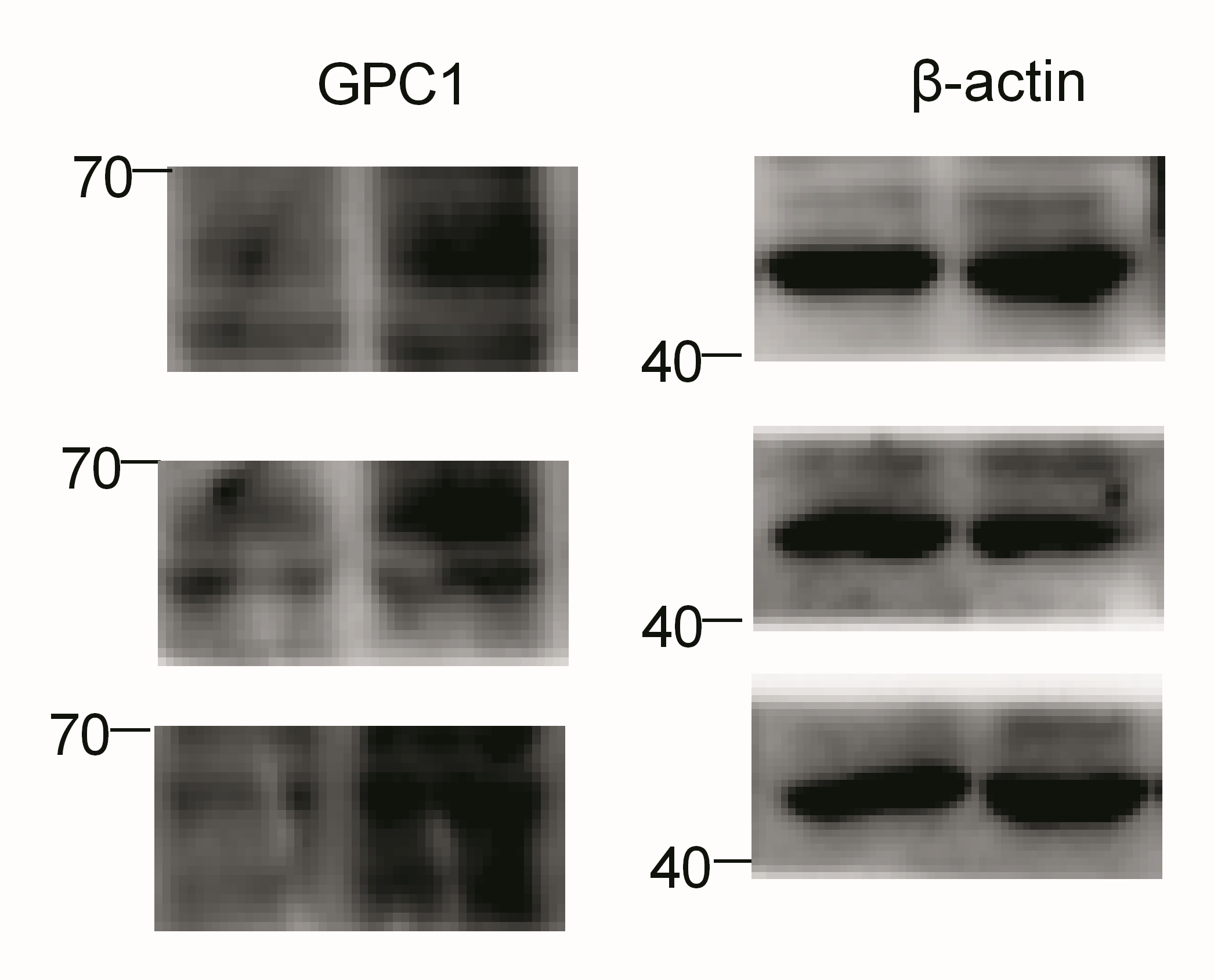


Fig.S2D


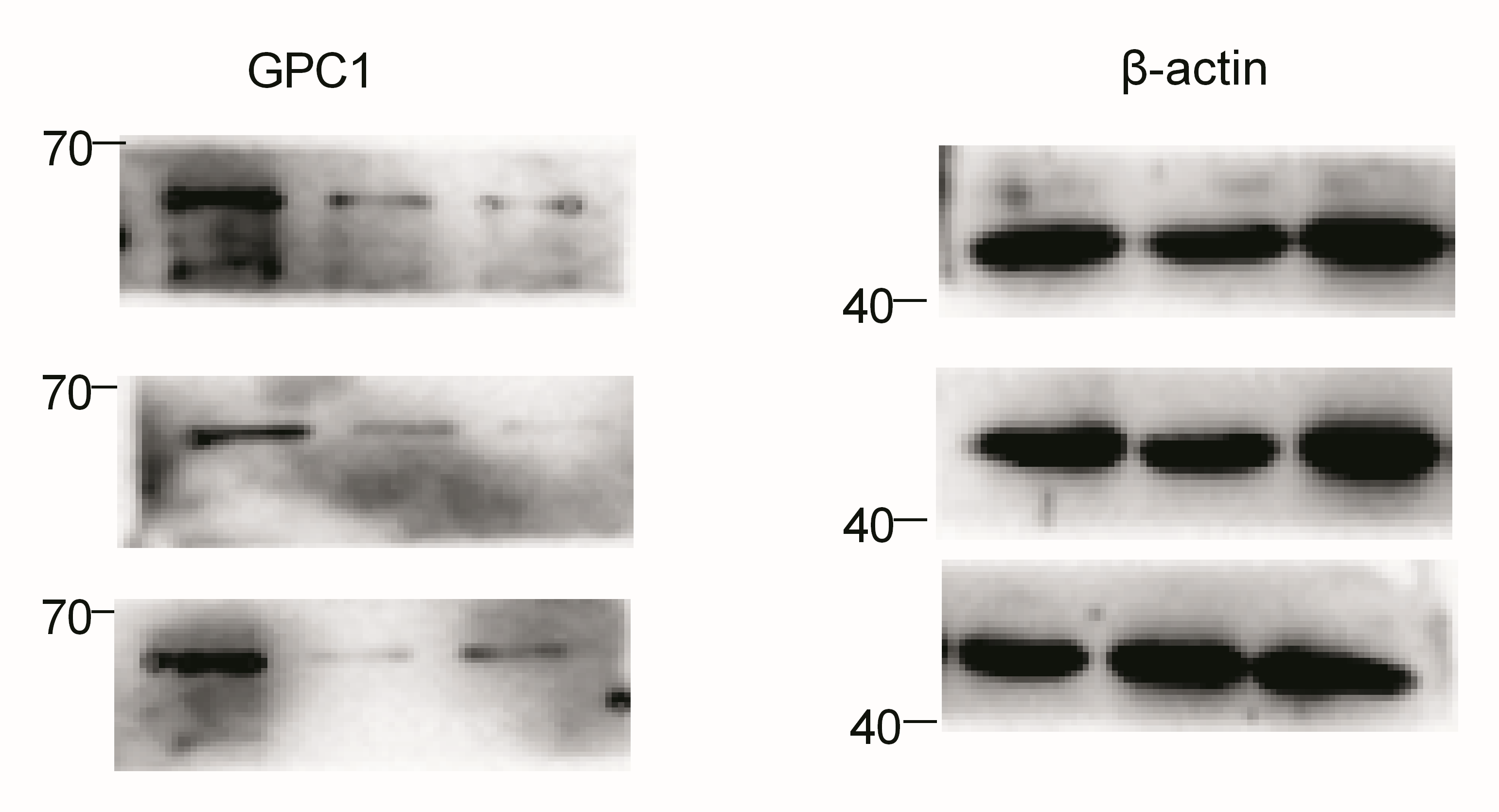

Supplement: Supplementary file 1 — supplemental materials of the manuscript [file 41419_2021_3547_MOESM1_ESM.doc]
